# Some Observations on the Diseases That Occurred on Board the Ship Europa, in the Service of the Hon. East India Company, during a Voyage from England to and from Madrass and Bengal

**Published:** 1794

**Authors:** John Watson

**Affiliations:** Late Surgeon of the Said Ship, and Now Surgeon at Wellingborough, in Northamptonshire.


III. *Some Obfervations on the Difeafes that occurred on Board the Ship Europa, in the Service of the Hon. Eaft India Company, during a Voyage from England to and from Madrafs and Bengal; by Mr.* John Watfon, *late Surgeon of the faid Ship, and now Surgeon at Wellingborough, in Northamptonfhire. Communicated to Dr.* Simmons, *by* John Lorimer, *M. D. Fellow of the Royal College of Phyficians of Edinburgh, Phyfician to the Army, and to the Hon. Eaft India Company.*

THE difeafes which prevail in the beginning of Southern voyages, have been fo often often pointed out, that I ſhould have thought it quite unneceſſary to offer any obſervations on the ſubject, if we had met only with the common prediſpoſing cauſes to combat with ; but this, I have great reaſon to believe, was not our caſe.

A fever, which we carried out with us, joined its influence to the common inflammatory fever, which is almoſt always met with upon approaching a warm latitude in a crowded ſhip, and I believe was the chief cauſe of our continuing a ſickly ſhip during the voyage. The caſe I allude to, as the cauſe of this miſchief, is that of Samuel Hall, one of the ſhip's company, who had been ill ſome days before we left Graveſend *, with ſymptoms of fever, for which, in the beginning, he had little medical aſſiſtance. When I firſt ſaw him, which was not till about the tenth day from the commencement of the diſeaſe, he had a quick ſmall pulſe, with coſtiveneſs ; a dry and parched tongue ; hot and dry ſkin ; and nauſea. This man recovered, but continued, for ſeveral weeks, in a ſtate of convaleſcence ; and, during this

* The Europa ſailed from Graveſend in January, 1792 and returned to England in April, 1793.

 time, time, a ſimilar fever attacked every man in the birth he was in, and it was very obſervable, that wherever it ſpread, the whole of the birth or meſs, in which it made its appearance, were more or leſs affected with it. We were now approaching the line, and with nearly two hundred recruits on board, (all of them young men, unaccuſtomed to hot weather, and, in general, of plethoric habits) could not expect to be free from the complaints which uſually occur in ſuch latitudes, and more eſpecially in a crowded ſhip.

A fever began to ſpread itſelf among them, attended with the uſual ſymptoms; viz. an increaſed action of the heart and arterial ſyſtem, great thirſt, nauſea, a hot dry ſkin, and, generally, coſtiveneſs.

This diſeaſe, in the firſt inſtances, eaſily yielded to the common antiphlogiſtic plan; but as we advanced, I found, to my great mortification, that our number of ſick continued to increaſe; and that the fever, which at firſt had been merely inflammatory, now put on the appearance of a remittent, and was clearly epidemic. The recruits, from their ſituation on the orlop deck, were more affected than the ſhip's company, company, who breathed a purer air, though the latter by no means efcaped its effects*.

To render this fituation more tolerable, as many as could be accommodated were brought upon the gun deck, where a platform was erected for them, and every mode that could be devifed to remove the foul air from below, was made ufe of. Cleanlinefs, of courfe, was the firft ftep; and the next was, to dry up thofe hot moift vapours, which are allowed to be fo baneful, not only to the fick, but even to the moft robuft and healthy. Fumigations, and wafhing with vinegar, were daily made ufe of; but the means I principally depended on, and which had the defired effect, confifted in having ftoves on the lower deck, that were gradually moved fore and aft, and occafionally fprinkled with nitre. The fire had the good effect of drying up the moift vapours,

* On the voyage Outwards, the fhip's company confifted of 103; of thefe, 86 were fick, and only one died; This death was in confequence of a fall. The recruits and paffengers from England were 158, to which were afterwards added, from Madrafs to Bengal, 270 more, making in the whole 428; and of thefe, 264 were on the fick lift, but only one of them died.

and the nitre ſupplied the ſick with good air. For a hint upon this ſubject, I am obliged to Dr. Lorimer, to whom, I am ſure, it will give pleaſure to be informed that it was attended with very ſalutary effects.

The fever, as I have before obſerved, changed its type, as we advanced to the ſouthward. Its general mode of attack was with rigors, head-ach, giddineſs, nauſea, and vomiting of bile; a quick but not a hard pulſe, ſometimes fluttering and unequal; great pain in the loins, with laſſitude; a dry parched ſkin; eyes full, heavy, and yellow; and great thirſt. On the firſt attack I ordered ſome of the antimonial emetic mixture * to be given; and I obſerved, that, in general, the patient was relieved by it, whether a vomiting of bile was the conſequence or not, for that was not always the caſe. And I conceived, that ſome irritating matter (which perhaps had been ſwallowed with the ſaliva, and cauſed the nauſea) was removed by the vomiting. An aperient antimonial mixture † was then

* *Miſtura Antimonialis Emetica.*

℞. Antimonii Tartariſati, gr. xvj

Aquæ Puræ, ℔i. M.

† See the note in page 32.

given, given, until it had operated by ftool, by which time, generally fpeaking, a gentle perfpiration had taken place, and then the bark was immediately adminiftered in as large dofes as the ftomach would bear. The very early exhibition of this valuable medicine, I think, never had a fairer trial, than I had an opportunity of giving it; and the fuccefs was equal to my moft fanguine expectations, for in no one cafe did it fail, although I had, at one period of the voyage, upwards of feventy patients making ufe of it, and that for a confiderable length of time, having had a conftant fucceffion of them. One patient only died, viz. J. Thompfon; but even in this inftance the failure, I am of opinion, ought not to be afcribed to the bark. The cafe itfelf was attended with fingular circumftances, and as it ended fatally, I have tranfcribed it from my diary, and fhall infert it at the end of my paper *, with the addition of one of the fuccefsful cafes †, in order to give a more perfect idea of the nature of the fever.

If we, for a moment, confider the fingle circumftance of fo many people confined in a

* See Cafe I. page 38.

† See Cafe II. page 41.

fmall fmall place with fevers, it will not appear extraordinary that one fhould die, but that fo many fhould recover; for during our paffage to Madrafs, three hundred and fifty perfons were at different times affected.

In general, the bark was given within thirty-fix hours after the firft attack; very often in lefs, and feldom later than forty-eight hours. If the ftomach retained the firft two or three dofes, (which were given hourly) it generally brought on a diftinct remiffion; and in four or five days the patients were left in a convalefcent ftate; and then the bark was adminiftered (but in lefs frequent dofes) with wine. To recover them from this convalefcent ftate, required fome attention; and their diet was regulated with as much care as poffible; for although our powers were very limited, yet it was found that much might be done by attention, and that the different provifions which are allowed, might be fo managed, as to render them much more conducive to health than the general mode of ufing them is, and, at the fame time, quite as grateful. Salted provifion, for men in their fituation, was evidently very improper; but unlefs we could fatisfy them with fomething elfe, it was impoffible to prevent their ufing it. For this purpofe,

I or-

I ordered a ſoup to be made, by cutting up and boiling one piece of ſalt beef, which had been previouſly waſhed, in a great quantity of water, and afterwards thickening it with oatmeal and barley. This made a tolerably grateful and freſh ſoup; at leaſt it gave ſo much the appearance of ſoup to the gruel, as to induce the patients to eat it with ſatisfaction; and I had the pleaſure of ſeeing them recover by this kind of management.

The neceſſary attention to ſo many people, could not fail, as may be eaſily conceived, to take up the time of myſelf and aſſiſtant (Mr. Walker) very completely. There was, indeed, ſcarcely a moment in which one or both of us were not employed in viſiting the ſick, in adminiſtering medicines to them, or in attending to other duties equally neceſſary. By other duties, I mean the attending to the execution of every plan which could be conducive to cleanlineſs, good order, and a free circulation of good air; for I cannot help conſidering theſe as the firſt and great cauſes of health, or diſeaſe, on board a ſhip, and without them medicine will avail but little. Theſe avocations, as I have juſt now obſerved, ſo completely employed us, that that it was impoffible to be very minute in each day's report. Any material alteration was, indeed, noticed in the diary; but in general, only the ftate of the fever, and medicine given, were noted. Both myfelf, and Mr. Walker (whofe great affiduity and attention to the fick I fhall ever remember) frequently felt the effects of the fever; for my own part, I never, for fome time, came up from vifiting them, that I did not experience headach and a quickened pulfe. The only precautions I made ufe of, confifted in keeping my ftomach and bowels clear, and in taking occafionally a little bark. By thefe means I lived, as it were, in the midft of fever for three months, without experiencing any other effects of it than thofe I have juft now mentioned; and Mr. Walker, who adopted a fimilar plan, was equally fortunate.

We ftopped at St. Jago, one of the Cape de Verd iflands, with the hopes of procuring a fupply of fruit and other refrefhment; but here we met with a difappointment, owing to a drought which had prevailed in the ifland during three or four feafons, fo that we left it without deriving much advantage from our vifit.

From thence we proceeded to Madrafs, without out ftopping any where, and had rather a tedious paffage; the fever gaining ground in point of numbers all the time, but without being attended with any fatal confequence, except in the fingle cafe already related.

In the month of June, (at the latter end of which we arrived at Madrafs) feveral of our convalefcents were attacked with fcurvy, but not in any very material degree; the very few antifcorbutics we had, were made ufe of, and the difeafe was kept under till our arrival, when the fick were all fent afhore, and remained there till the 18th of July, when they again embarked for Bengal, with a detachment of His Majefty's 76th regiment.

During our ftay at Madrafs, every means were made ufe of to remove, if poffible, difeafe from the fhip, by wafhing and fumigating every part of her. But our fhip's company ftill continued fickly, fo that on our arrival at Diamond harbour, the fick lift was upwards of twenty. The unhealthy fituation of this place is well known; but the hofpital, though placed on fo unhealthy a fpot, has all the advantages that could be given to it. It is raifed confiderably, and ftands upon arches; and the ground near it is drained as much as poſſible; but ſtill it is a marſhy ſituation, and the patients cannot fail to be affected by the low ſwampy grounds which ſurround it for ſome miles.

Notwithſtanding it was not ſo eligible a place as could be wiſhed, it was thought more adviſable tc have our ſick there than in the ſhip, for two reaſons; firſt, for the ſake of ſeparating the ſick from the healthy; and ſecondly, to give an opportunity of effectually cleaning the ſhip. Thoſe who were removed from the ſhip to the hoſpital, (which at this time was empty, ſo that we could give them large accommodation) very ſoon benefited by the change; others, who were taken ill whilſt we remained there, were not ſo fortunate, as their complaints were in general aggravated by their intemperance. Their principal complaints were fevers of the remittent or marſh kind, attended with bilious vomiting and purging, and great tendency to dyſentery. In moſt of theſe caſes there was a greater or leſs tendency to delirium, in proportion as the patients were more or leſs of a plethoric habit. In ſeveral of theſe the pulſe was ſo full and hard, that I ſhould have been induced to take ſome blood from them, had I not feen the ill effects of it, even in very fmall quantities, during a former voyage, under fimilar circumftances. In general, the early exhibition of the bark was very effectual, provided proper attention was paid to the ftate of the ftomach and bowels. The aperient antimonial mixture * was what I principally made ufe of to clear the ftomach and bowels, and in general it had the good effect of doing both, and, at the fame time, excited a gentle diaphorefis; the bark was then given freely, but, I obferved, if it had not a good effect in a few days, the difeafe became obftinate, and generally ended in vifceral obftruction; to remove which it was neceffary to ufe mercury, which feldom failed to produce the defired effect. Thefe cafes were alfo frequently caufed by relapfes, in confequence of thofe irregularities which it is impoffible to prevent feamen committing.

In the month of September, dyfenteries, accompanied with fever, were frequent. In many of thefe cafes, alfo, after proper evacuations, the bark was given freely. In fome other cafes of dyfentery, I found the ufe of diapho-

* See the note in page 32.

retics retics very efficacious. This mode of treatment conſiſted in giving ſuitable doſes of the antimonial aperient mixture* during the day, which ſometimes excited vomiting, but always cauſed copious ſtools, and generally brought on a gentle diaphoreſis; this was encouraged by warm diluting liquors, and in the evening a pill was given, compoſed of one grain of opium, and gr. ⅛ of antimon. tartariſat. In the morning the mixture was again had recourſe to, and this plan was continued till the bowels were relieved, and the ſtools were become more natural, after which the antimonial opiate only was given.

Upon leaving Bengal, to return to Europe, our complaints were principally intermitting fevers, the remains of the marſh fever, which had taken that type; dyſenteries; viſceral obſtructions; and inflammations, principally of the

* *Miſtura Antimonialis Aperiens.*

℞. Antimonii Tartariſati, gr. ij.

Mannæ, ℥ſs.

Cremor. Tart. ℥ij.

Kali Tartariſati, ʒiij.

Spir. Æther. Nitr. ʒij.

Aquæ Puræ, ℔ſs. M.

liver, liver, but often of the fpleen and mefenteric glands.

Every opportunity I have had of obferving the difeafes of hot climates, confirms me in the opinion I have long held, in common with many other medical men, that the great fource of health or difeafe, in hot climates, is centered in the natural or difeafed condition of the liver; and that every chronic difeafe arifes, in a confiderable degree, from fome defect of that vifcus. In many acute diforders, alfo, this organ has its fhare; and in every kind of ficknefs, whether local or general, that is peculiar to hot climates, it is material to examine it; for no perfect cure, I am perfuaded, can be made, or a relapfe prevented, without having a ftrict eye to it. Fluxes are frequently caufed by obftructions of this vifcus, and however they may be palliated, can never be cured, without firft removing the fource of the difeafe, in the liver, which, I believe, is only to be done by mercury.

In our voyage outwards, feveral of our men had frequent relapfes of fever, attended at times with a flux, which I never could totally remove, till I joined mercury with the bark. This proved efficacious in promoting good fecretions,

 and and confequently more natural ftools; and the fever afterwards gave no trouble. In all thofe patients I found the urine much affected, as, indeed, it is in all cafes where bile prevails.

A long-continued ufe of the bark has been faid to occafion thefe complaints; but, I think, with great injuftice. It is certain, that we cannot remove the caufe of the fever or flux by means of the bark alone; but in the many cafes in which I have given it, combined with mercury, I never could perceive any ill effect from it; on the contrary, the difeafe has yielded to their united powers, and I have thought that the patient was lefs reduced, than by ufing mercury alone.

In fluxes only, proceeding from obftructed liver, the bark will feldom be neceffary; but in fuch cafes, if there be a difpofition to fever, it will generally be found ufeful, more particularly fo if given a day or two before the fpring tides; for at thofe periods the difpofition to fever will always be found to increafe, and confiderable advantage will arife from attending to this circumftance. I experienced this, not only whilft we were lying in Diamond harbour, but during the whole paffage from Bengal to the Cape of Good Hope.

As we advanced to the Southward, the ſcurvy began to make its appearance on board, more particularly among the convaleſcent, and thoſe who had lately recovered from fevers.

If we reflect a moment upon a diet ſo deprived of nutritious powers, as ſalt beef and biſcuits, with a ſmall allowance of water, we cannot be at a loſs to account how eaſily ſuch a diſeaſe as the ſcurvy might ariſe in the habit, even of thoſe who had before enjoyed health, much more ſo of thoſe whoſe blood had been impoveriſhed by diſeaſe. And here I cannot but lament, that there is not a more liberal allowance of thoſe things, which have been found to be ſo beneficial in counteracting the effects of this dreadful diſeaſe; for as the cauſes, before mentioned, are not the only pre-diſpoſing ones, but are much aſſiſted by a moiſt cold air, it naturally follows, that upon a change of air we have not ſo much to combat with.

Inſtances I have repeatedly ſeen, where the ſcurvy has attacked numbers in a ſhip going round the Cape of Good Hope; but upon ſtanding to the Northward, and having a clear dry air, with ſtrict attention to their diet, cleanlineſs and exerciſe, and giving as largely of the antiſcorbutic proviſions as could be al-

 lowed, lowed, the difeafe has been removed. And when it is confidered, that by proper management it would be attended with very little, if any, additional expence, to put on board a fufficient quantity of fuch provifions, it leads me to hope, that it is only neceffary to point them out, in order to their being adopted. The known humanity and liberality of the owners in general, warrant fuch a fuppofition.

If they would take the trouble of examining any of their furgeons upon this fubject, who have been long enough in the fervice to experience the want and to fee the utility of thefe things, I doubt not but fuch regulations would take place as would be very gratifying to a humane mind, and beneficial to the fervice in general.

With refpect to the mode adopted for the relief of thofe who were affected with this complaint, I have only to obferve, that a diet was formed as void of putrefcent things as poffible. They had plenty of acidulated liquors, and were recommended to ufe as much exercife as poffible. The only alarming fymptom was a dyfpnœa, which many of them were affected with, and which I found was greatly relieved by camphor, given in the form of a bolus. This was almoft the only medicine I gave them; it generally caufed a gentle perfpiration, and relieved their breathing.

Upon our arrival at the Cape of Good Hope, on our voyage homewards, the fick were plentifully fupplied with vegetables and foup, which foon recovered them. A few of them remained in a convalefcent ftate for a little time; but the very ample allowance of frefh provifions, which was ferved out for a confiderable time afterwards, removed every appearance of fcurvy, and we became at length a healthy fhip; fo that after we left St. Helena, we fuffered very little inconvenience from difeafe.

We were fo fortunate as to meet with few accidents that required chirurgical affiftance; and thofe we did meet with were attended with no circumftances worth relating; therefore I have not thought it neceffary to mention them in this brief account of the difeafes we had to encounter with.

### CASE I.

May 24th. John Thomſon, aged thirty years, was attacked with ſymptoms of fever, viz. a quick full pulſe; giddineſs; great headach; nauſea and vomiting; heat and dryneſs of the ſkin, and great thirſt. He was directed to take the emetic antimonial mixture *.

25. The emetic brought up much bile; the headach was now leſs violent; body coſtive; other febrile ſymptoms as yeſterday. He was ordered to take of the aperient antimonial mixture † every two hours, till a ſtool ſhould be procured.

26. Symptoms as yeſterday. Medicines repeated.

27. Fever continued, with coſtiveneſs. The mixture was repeated, and a purgative clyſter adminiſtered. Stools being procured by theſe means, recourſe was had to the following mixture, of which, from an ounce to an ounce and a half, was given every hour.

* See page 24. † See page 32.

℞. Pulv. Cort. Peruvian. ℥iiſs.

Tincturæ ejuſdem ℥iij.

Aquæ Puræ, ℔ij. M.

28. A ſlight remiſſion; pulſe better; body natural. The bark mixture was continued.

29. He was free from fever all night, and continued the uſe of the bark till about four o'clock, P. M. when a ſudden proſtration of ſtrength took place; the pupils of the eyes were much dilated, and he appeared comatoſe; but his pulſe continued good, and he perſpired freely. Towards midnight, however, his pulſe ſunk conſiderably. A bliſter was applied between his ſhoulders.

30. He continued comatoſe, but ſeemed at times ſenſible; refuſed every thing offered to him; his pulſe was rather quicker; the bliſter roſe.

June 1. He appeared to be more ſenſible, but his pulſe was quick and low. He took liberally of a camphorated mixture, decoction of bark, and Madeira wine.

2. He was much the ſame as yeſterday. The ſame plan of treatment was continued.

3. The ſymptoms, in general, were worſe; he had taken very little medicine during the

 night, night, and ſwallowed what he did with difficulty. Took his medicines and Madeira wine alternately; his extremities were cold; ſinapiſms were applied to the feet.

4th, 5th, and 6th. No material alteration.

7. Continued in the ſame ſtate. As he had not had a ſtool for ſome days, an enema was given, and his medicines and wine were ordered to be continued.

8. The enema procured ſeveral copious and very fetid ſtools. He appeared more ſenſible; took his medicines and wine in the night.

9th and 10th. On each of theſe days was free from fever, but continued the uſe of his medicines.

11. In the ſame ſtate; refuſed his medicines.

12. Very low, but appeared more ſenſible; refuſed every thing that was offered to him.

13. Refuſed his medicines, but took wine frequently; ſome livid blotches appeared over the trochanter major, os ſacrum, and lower vertebræ lumborum. Had a very fetid ſtool this morning.

14. Pulſe very feeble.

15. Pulſe continued to ſink all night, and he expired about four, A. M.

In

In this cafe the bark was not given quite fo early as in the generality of the other cafes, owing to an obftinate coftivenefs during the firft three days of the fever ; but when it was given, it appeared to have a good effect. I am unable to account for the fudden change in this cafe, at a time when I was beginning to flatter myfelf that the patient was out of danger. Had this change taken place fooner, it might by fome, perhaps, have been imputed to the evacuations by ftool, which were rather copious, but they had taken place two days before, and the patient had been free from fever that day, and had a tolerable pulfe ; but from the moment the fatal alteration commenced, the pulfe began to fink, and continued to do fo till he expired.

### CASE II.

April 11, 1792. Lat. 13. 52. S. Long. 30. 46. W. James Armand, aged twenty years, and rather of a full habit, was taken with the ufual train of febrile fymptoms, as headach, great pain in his loins, and naufea. He was ordered an antimonial emetic.

12. The

12. The emetic operated well. The ſymptoms of fever continued. Four grains of pulv. antim. were directed to be given every two hours.

13. The medicine had operated both upwards and downwards. The nauſea and febrile ſymptoms were as before. The antimonial was repeated, and after it had operated, the bark mixture was given frequently.

14. The febrile ſymptoms were abated. The uſe of the bark was continued.

15. He had an increaſe of fever in the night. The bark was continued.

16. He had leſs fever. The ſame medicine was continued.

17. The ſymptoms were about the ſame as yeſterday. He continued the uſe of the bark.

18. He was free from fever. The bark was continued.

19. He had a ſlight return of headach, with nauſea, and took ſome of the aperient antimonial mixture.

20. The antimonial brought up much bile, but procured no ſtool. He had an exacerbation of fever in the evening. The bark mixture was repeated frequently.

21. He had leſs fever. The uſe of the bark was continued.

22. Symp-

22. Symptoms the fame as yefterday, till the evening, when he had a return of ficknefs and fever. The antimonial aperient mixture was ordered to be repeated on the morning of the 23d.

24. The medicine had operated freely. He had a remiffion this morning, and continued to take the bark mixture frequently.

25. He had fome fever in the night. The fame medicine was continued.

26. He was free from fever.

From this time to the 30th, he continued to take the bark with wine, and perfectly recovered.

In this cafe nothing particular occurred diffe-rent from the general run of fevers, which we had at this time, except a greater difpofition to bile, than I generally met with. This made it neceffary to continue the antimonials longer, and even to recur to them after giving the bark.

*Wellingborough,*

*July* 1, 1793.

